# Dissecting the role of KLF5: from tumor progression to immune interactions with emphasis on glioma and bladder cancer

**DOI:** 10.3389/fimmu.2025.1730356

**Published:** 2026-01-08

**Authors:** Ze Yuan, Minyi Situ, Yimeng Ye, Jinhui Zhang, Kuntai Jiang, Shingyik Zhang, Xinpei Deng, Zhenqiang He, Juncheng Luo, Yanjun Wang

**Affiliations:** 1 State Key Laboratory of Oncology in South China, Guangdong Provincial Clinical Research Center for Cancer, Sun Yat-Sen University Cancer Center, Guangzhou, China; 2 Zhongshan School of Medicine, Sun Yat-sen University, Guangzhou, China; 3 Department of Neurosurgery, The Seventh Affiliated Hospital of Sun Yat-sen University, Shenzhen, China

**Keywords:** KLF5, multi-omics, pan-cancer, prognosis, tumor microenvironment

## Abstract

**Background:**

Krüppel-like factor 5 (KLF5) is involved in various aspects of tumor development, metastasis, and drug resistance through their regulation of transcription and translation, yet its functions in a comprehensive cancer framework are still unclear.

**Methods:**

Our research involved a detailed pan-cancer analysis using multi-omics data sourced from various public databases. We investigated the clinical characteristics, prognostic significance, mutations, and methylation patterns of KLF5 across various cancer types.

**Results:**

We discovered that KLF5 is implicated in tumor progression and are prognostic markers across pan-cancer. KLF5 is significantly linked to various malignant pathways across different types of cancer. Additionally, KLF5 has associations with several immune-related features. Ultimately, experiments were carried out to investigate whether KLF5 could serve as a promising indicator for glioma and bladder cancer.

**Conclusion:**

KLF5 may be utilized as a diagnostic tool for cancer, a predictor of its progression, and a guide for treatment., with particular promise as a therapeutic target for glioma and bladder cancer.

## 1 Introduction

Cancer still represents a considerable health challenge, despite significant improvements in diagnosis and therapy over the past decades (1). Increasing cancer rates and deaths are linked to population growth and aging, variations in risk factor prevalence and distribution, and socioeconomic advancements (2). Consequently, identifying new biomarkers and molecular indicators is essential for predicting patient outcomes and tailoring treatment strategies.

Krüppel-like factor 5 (KLF5) is a member of the KLF family, which is defined by a conserved C-terminal zinc finger domain that confers DNA-binding specificity (3–5), and whose members function as master regulators of fundamental cellular processes including proliferation (6), apoptosis (7), angiogenesis (8), and tumorigenesis (9–11).Existing research confirms that KLFs act as a regulator, playing both driving and inhibitory roles in key steps of progression and metastasis across multiple cancers (11–14).

Consistent with the functions of numerous KLFs, KLF5 exerts a context-dependent influence in oncology. Its well-characterized oncogenic activities are largely attributed to its capacity to transcriptionally activate a network of genes that fuel malignant cell growth (15) and metastatic spread (12). As a potent driver of tumor progression and metastasis, KLF5 accelerates the cell cycle (16), suppresses apoptotic signals (17), and promotes epithelial-mesenchymal transition (18) to enhance invasive potential. It regularly interacts with important oncogenic signaling pathways, including Wnt/β-catenin, to help maintain the properties of cancer stem cells (19). This pro-tumorigenic role of KLF5 is prominently observed in malignancies like lung cancer (18), colorectal cancer (19), liver cancer (16). However, KLF5 exhibits a context-dependent role in certain cancer types, notably in breast cancer (10, 15, 20–22) and prostate cancer (23–25). Studies utilizing multi-omics datasets to systematically investigate the role of KLF5 expression in pan-cancer contexts are still limited.

With the rapid development of omics detection methods such as high-throughput sequencing technology, multi-omics technologies including transcriptomics, genomics, proteomics, and metabolomics have become the core tools for analyzing the molecular profile of tumors (26, 27). Multi-omics technologies can systematically capture key information such as genetic variations, gene expression, protein regulation, and metabolic remodeling of tumor cells at different molecular levels, providing multi-dimensional data support for identifying tumor drivers and identifying potential therapeutic targets (28–30). Based on this, the emerging pan-cancer analysis has broken through the barriers of single-cancer research. By integrating multi-omics data from different cancer types, it conducts common and differential analyses across cancer types (31–33). This not only enables the discovery of shared molecular characteristics and signaling pathways among different tumor types, but also identifies unique molecular markers specific to certain cancer types. Therefore, a comprehensive profiling of KLF5 function in pan-cancer is necessary to address.

**Figure 1:**
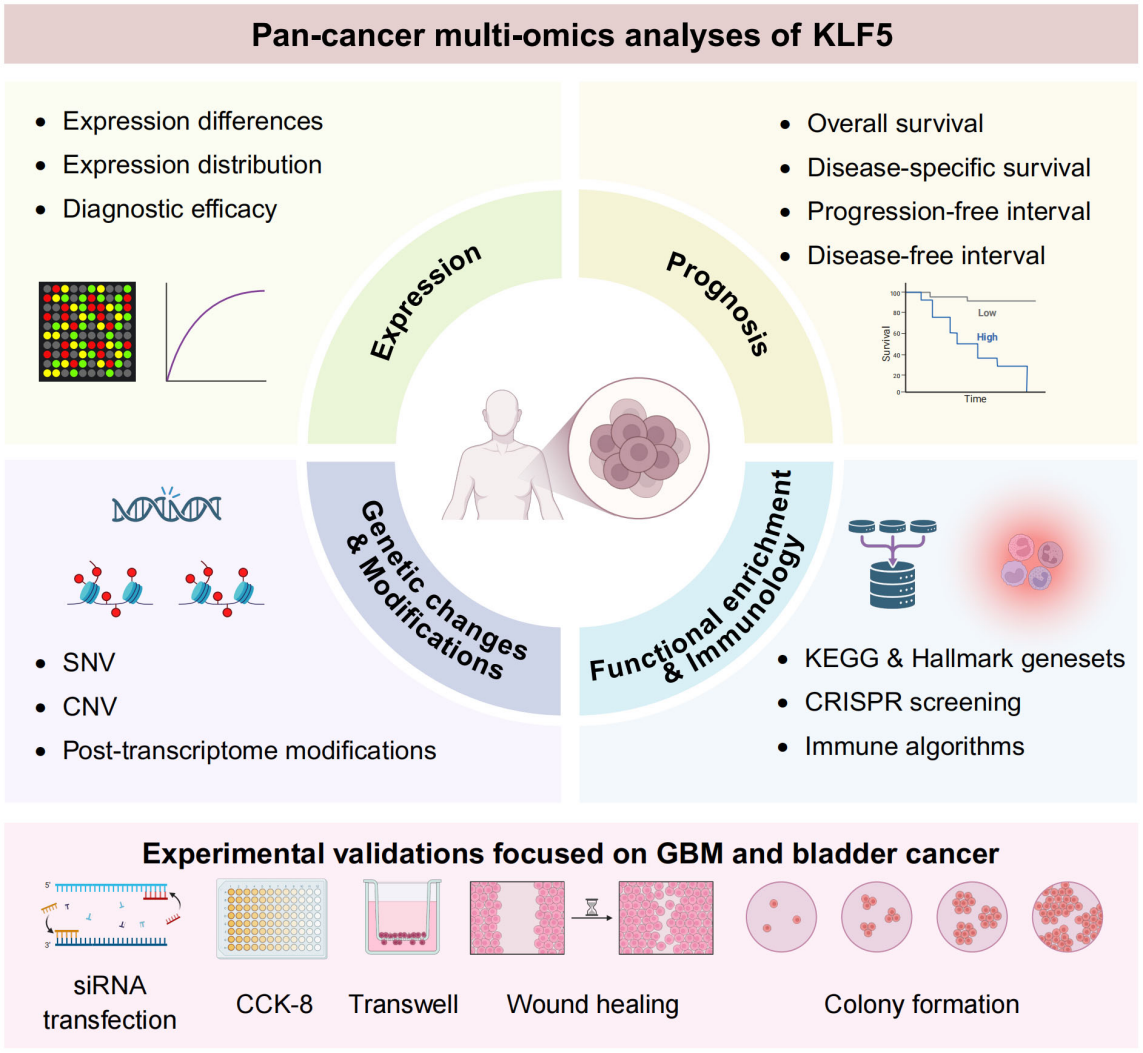
Flowchart of this study.

We conducted a comprehensive pan-cancer analysis of KLF5, examining its expression, prognostic impact, genomic alterations, post-translational modifications, and functional roles across multi-omics datasets. Validation through *in vitro* experiments confirmed that KLF5 is upregulated in glioblastoma (GBM) and bladder cancer, and that its knockdown effectively suppresses GBM and bladder cancer growth. These results indicate that KLF5 may be utilized as a diagnostic tool for cancer, a predictor of its progression, and a guide for treatment., with particular promise as a therapeutic target for glioma and bladder cancer. The main design of our study was shown in **Figure 1**.

## 2 Materials and methods

### 2.1 Data collection

We obtained RNA-sequencing profiles from the UCSC Xena database, which were normalized and log2-transformed, along with related clinical data from TCGA and GTEx. Detailed information of the enrolled samples was shown in **Supplementary Table S1**. The pan-cancer scRNA-seq datasets were sourced from the TISCH2 database (34). We leveraged the Gene Set Cancer Analysis (GSCA) database to investigate genetic changes within KLF5, including single nucleotide variants (SNV), copy number variations (CNV), methylation, as well as pathway activity (35). The external cohorts enrolled in this study were obtained from the BEST database (36).

### 2.2 Clinical outcome analysis

The ‘survival’ and ‘survminer’ R packages were utilized for a Kaplan-Meier (K–M) analysis to assess whether the KLF5 expression is linked to survival outcomes such as overall survival (OS), disease-specific survival (DSS), disease-free interval (DFI), and progression-free interval (PFI). To determine the optimal cut-off point, the ‘surv_cutpoint’ function was utilized, and the receiver operating characteristic (ROC) curve analysis was done using the R package ‘pROC’.

### 2.3 Immune cell infiltration analysis

We employed the ‘immunedeconv’ R package to assess immune cell infiltration data from the TCGA pan-cancer cohort (37).

### 2.4 Cell lines and cultures

The cell lines used in this study were obtained from the American Type Culture Collection. GBM cell lines (U251 and T98G) were grown in DMEM (Gibco, USA) and bladder cancer cell lines (SV-HUC-1, T24, 5637, and RT113) were cultured in RPMI1640 medium (Gibco, USA) supplemented with 10% fetal bovine serum (Gibco, USA) and antibiotics, maintained at 37 °C with 5% CO2.

### 2.5 Transient transfection

For RNA interference, small interfering RNAs (siRNAs) aimed at KLF5 and a non-targeting control were synthesized and acquired from GenePharma. Detailed primer sequences and siRNA sequences are listed in **Supplementary Table S2**, **S3**. Lipofectamine 3000 (Invitrogen) was used to transfect siRNAs into the cells, adhering to the manufacturer’s protocol.

### 2.6 *In vitro* experimental validations of KLF5

For CCK-8 assays, cancer cells were placed in 96-well plates, followed by the addition of CCK-8 solution to each well, and incubated for 2 hours to evaluate cell viability. OD450 values were tracked over a period of 5 days.

For transwell assays, enzymatic digestion was performed on cancer cells, which were then resuspended. Cells were placed in the upper chambers without fetal bovine serum (FBS), while the lower compartment with cross-pores had a medium containing 20% FBS. Following incubation period, the cancer cells that had migrated were fixed using methanol and stained with 0.1% crystal violet.

For wound healing assays, transfected cancer cells were grown in 6-well plates, and a 100 μL pipette tip was used to create wounds. Microscopic images were taken at the beginning and after 24 hours, with ImageJ software used to quantify the scratch area.

For colony formation assays, cancer cells were seeded in 6-well plates with medium containing 10% FBS. After two weeks, colonies were fixed with methanol and stained with 0.1% crystal violet, each for 20 minutes.

### 2.7 Statistical analysis

The statistical analyses in this research were conducted using R and GraphPad Prism software. Comparisons between two groups were conducted using the Student’s t-test and Wilcoxon test. Cox regression and log-rank tests assessed the prognostic importance of KLF5. The false discovery rate was corrected using the Benjamini–Hochberg method, with an adjusted p-value of under 0.05 indicating significance.

## 3 Results

### 3.1 KLF5 expression patterns and its prognostic value in pan-cancer

**Figure 2:**
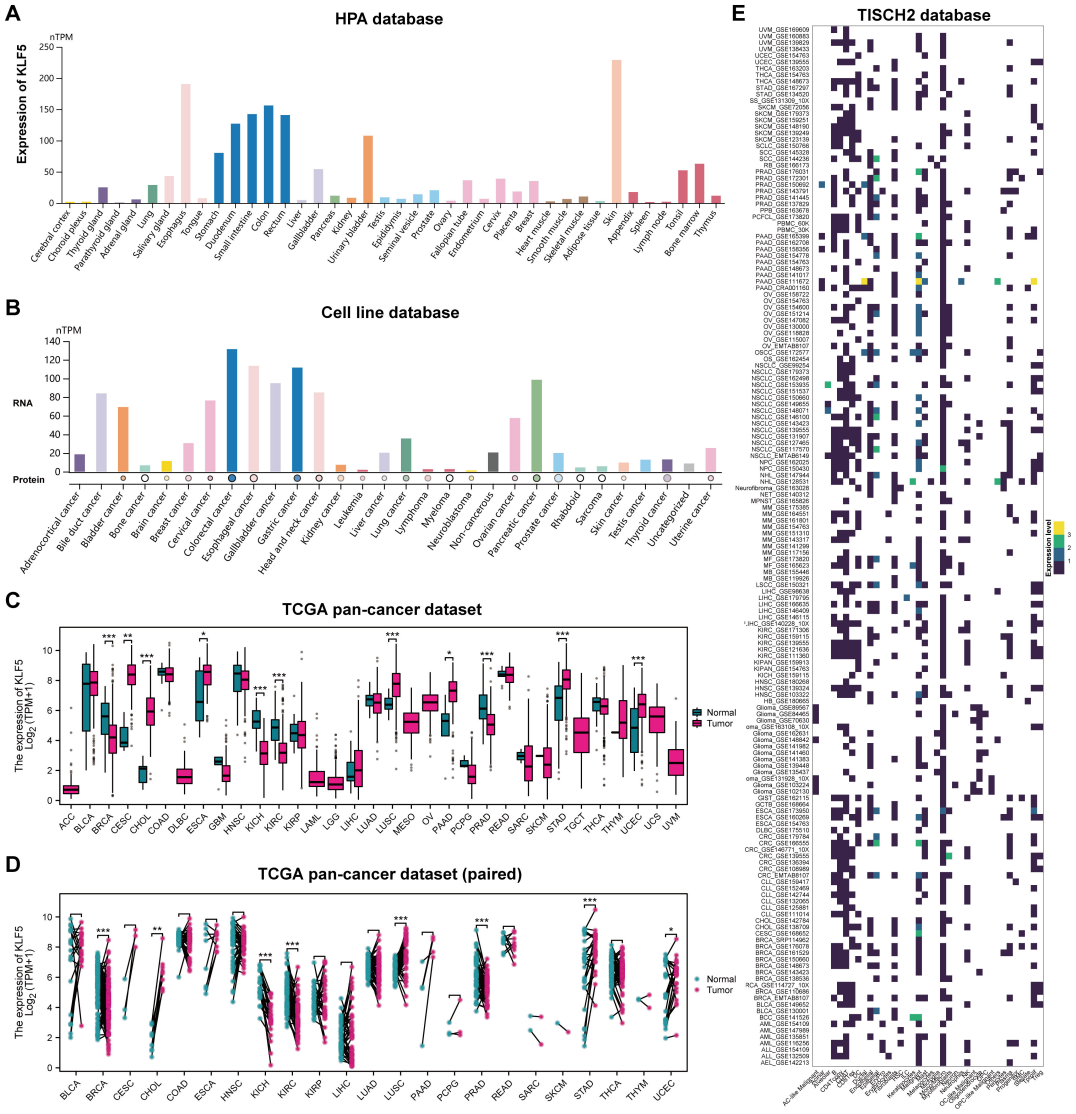
Tissue distribution and cancer-type specific expression profile of KLF5. **(A)** KLF5 mRNA expression across various normal human tissues based on the HPA database. **(B)** KLF5 expression levels across various cell lines. **(C)** Differential expression analysis of KLF5 between tumor and normal tissues across TCGA pan-cancer datasets. **(D)** Differential expression analysis of KLF5 between tumor and paired adjacent normal tissues across TCGA pan-cancer datasets. **(E)** Heatmap showing the expression level and distribution of KLF5 across pan-cancer datasets using TISHCH database. * means p<0.05, ** means p<0.01, and *** means p<0.001.

The Human Protein Atlas datasets (HPA) revealed a highly expression of KLF5 in different epithelial tissues including esophagus, bladder, stomach, duodenum, small intestine, etc. (**Figure 2A**). KLF5 transcription levels are elevated in the majority of cancer types, as revealed by transcriptomic analysis across various cancer cell lines (**Figure 2B**). Further analysis of the TCGA pan-cancer dataset demonstrated that significant differences in KLF5 gene expression between tumor tissues and their corresponding normal tissues were observed in both paired and unpaired samples for BRCA, CHOL, KICH, KIRC, LUSC, PRAD, STAD, and UCEC (**Figures 2C, D**). Cellular localization of KLF5 expression was analyzed using the TISCH2 pan-cancer scRNA-seq database, where it was found to be predominantly enriched in malignant and epithelial cells (**Figure 2E**). These results indicated that KLF5 possessed vital role in tumor progression.

**Figure 3:**
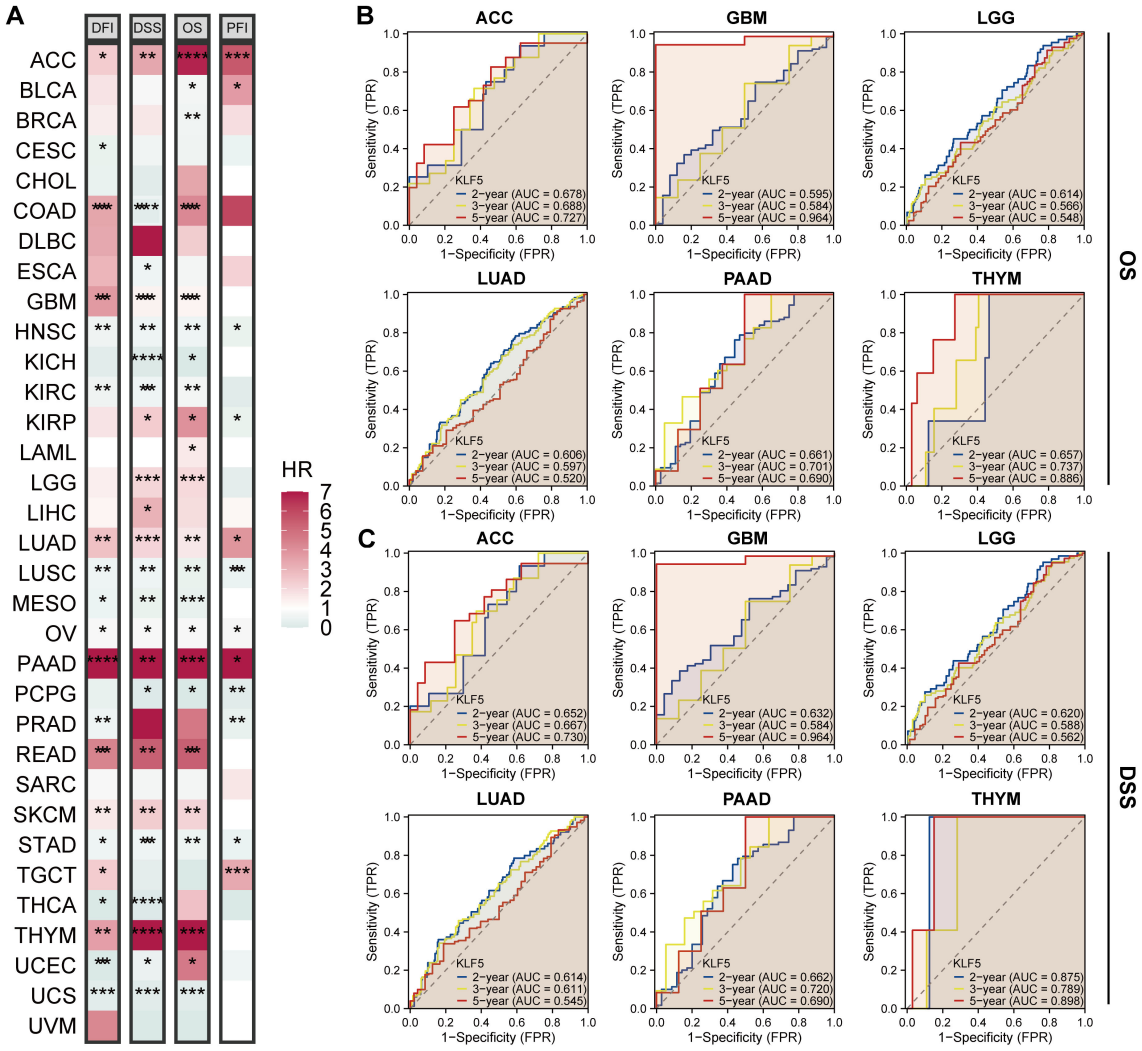
Prognostic significance of KLF5 expression in pan-cancer. **(A)** Heatmap showing the Hazard ratio (HR) of KLF5 based on overall survival (OS), disease-specific survival (DSS), progression-free interval (PFI), and disease-free interval (DFI) across multiple cancer types. **(B, C)** ROC curves showing the AUC values based on KLF5 expression in pan-cancer. * means p<0.05, ** means p<0.01, *** means p<0.001, and *** means p<0.0001.

Analyses based on TCGA database in the pan-cancer clinical prognostic significance of KLF5 were then performed. KLF5 expression was identified as a significant high risk prognostic factor in several cancers, including ACC, COAD, GBM, LGG, LUAD, PAAD, READ, SKCM, and THYM (**Figure 3A**). Furthermore, in a subset of these cancers (ACC, GBM, LGG, LUAD, PAAD, and THYM), KLF5 expression served as a predictor with high accuracy for both OS and DSS, as demonstrated by ROC analysis (**Figures 3B, C**).

### 3.2 Genomic and epigenetic alterations analysis of KLF5 in pan-cancer

**Figure 4:**
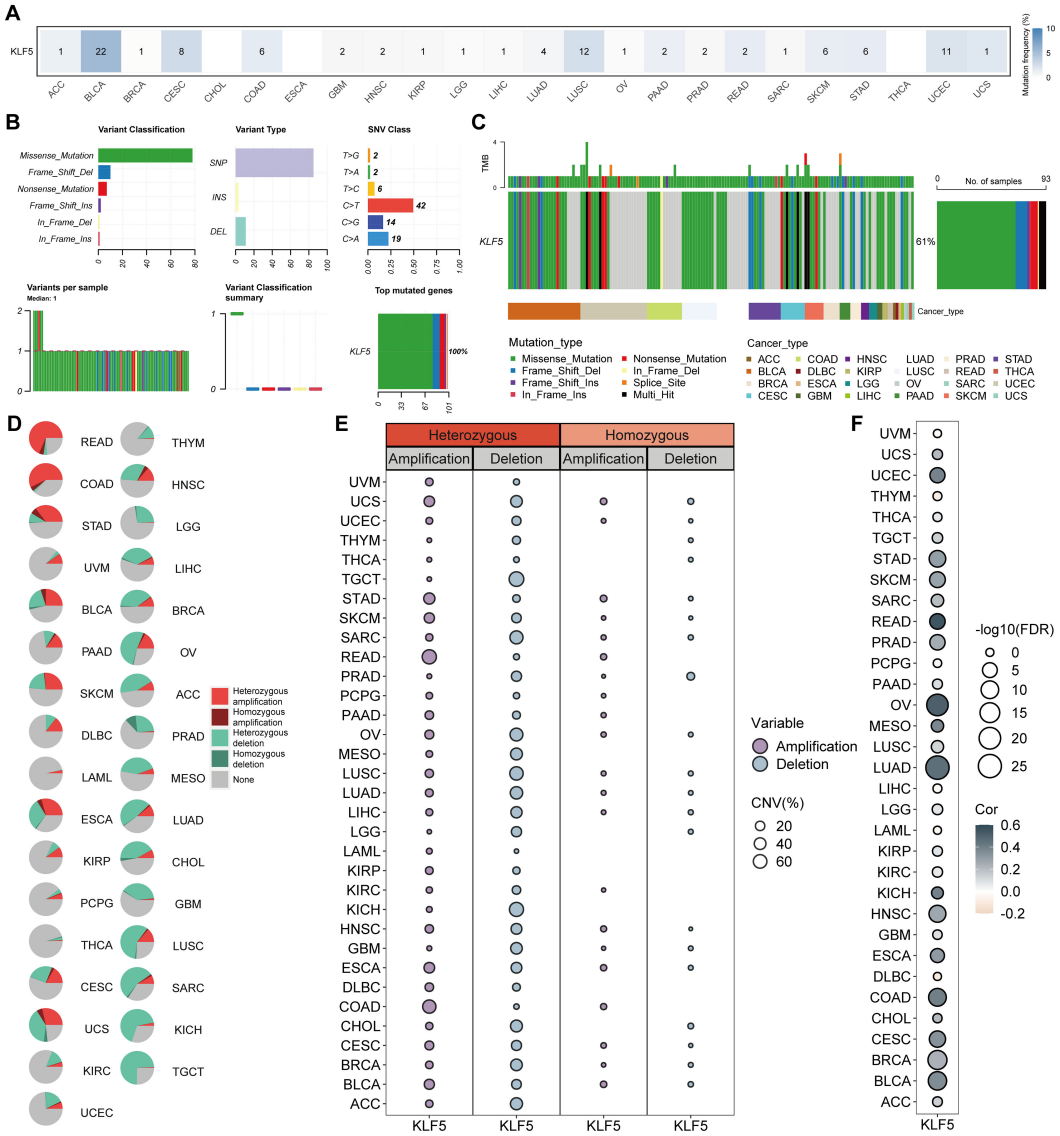
Genomic alterations of KLF5 in pan-cancer. **(A)** The SNV profile of KLF5 in each tumor type. **(B)** The SNV summary of KLF5 in pan-cancer. **(C)** Oncoplot of the mutation distribution of KLF5 in pan-cancer. **(D)** Pie charts showing the distribution of copy number variations (CNVs) of KLF5 among different tumor types. **(E)** Profiles of heterozygous and homozygous CNVs, including amplification and deletion rates, across cancers. **(F)** Bubble plots indicating correlations between CNVs and KLF5 mRNA expression levels (FDR-adjusted).

KLF5 was found to exhibit widespread SNV and CNV events across multiple cancer types. A high frequency of SNVs was observed in BRCA, CESC, COAD, LUSC, SKCM, STAD, and UCEC, predominantly characterized by missense mutations and single-nucleotide polymorphisms. A notable enrichment of C-to-T transitions was identified as a major component of the SNV profile (**Figures 4A, B**). Additionally, the assessment of the KLF5 mutation landscape in a pan-cancer context demonstrated that missense mutations were the most prevalent overall, yet the mutation types differed among specific cancer types (**Figure 4C**). Pan-cancer analysis of KLF5 copy number variation (CNV) patterns, revealed a predominance of heterozygous amplification in which READ, STAD, ESCA, BLCA, and COAD, while the predominance of heterozygous deletion in HNSC, LGG, GBM, BRCA, PRAD, LUAD, KIRC, TGCT and OV (**Figures 4D, E**). Additionally, the correlation analysis demonstrated that KLF5 CNV alterations were associated with a widespread positive correlation with its mRNA expression across most cancer types (**Figure 4F**).

**Figure 5:**
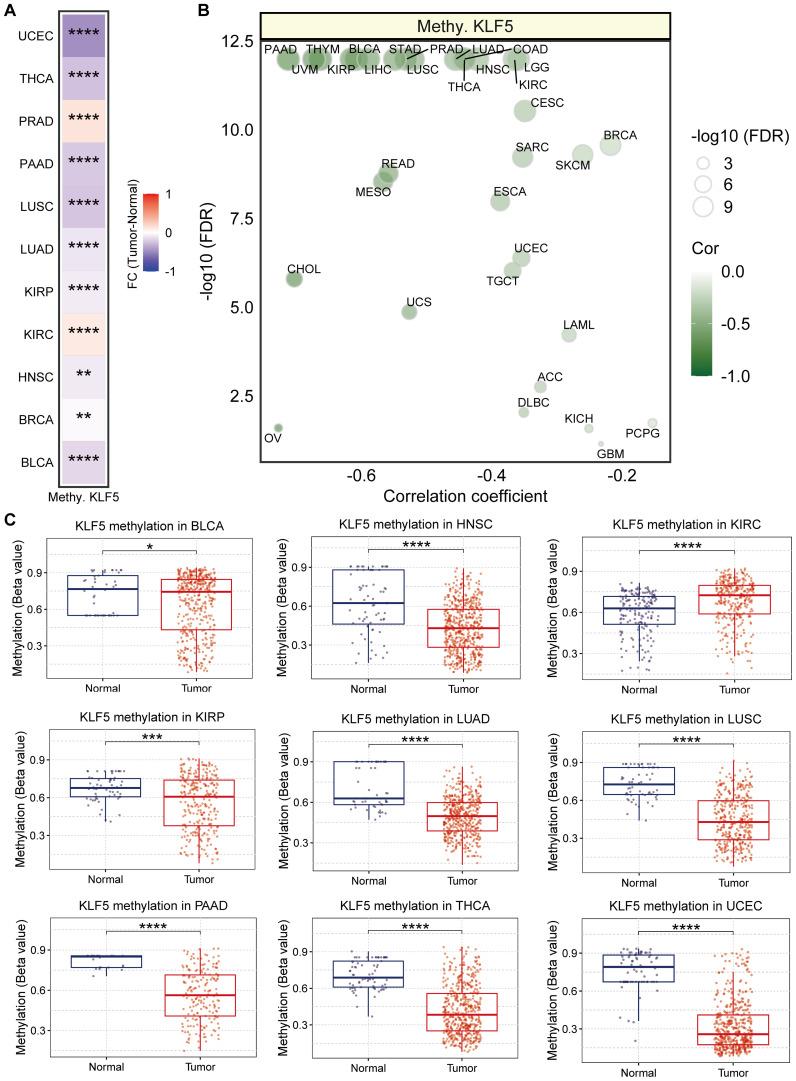
Methylation landscape of KLF5 in pan-cancer. **(A)** Heatmap showing the Fold change (FC) of KLF5 methylation values across pan-cancer. ** means p<0.01, and **** means p<0.0001. **(B)** Bubble plot illustrating the correlation between KLF5 mRNA expression and DNA methylation levels in various cancers. **(C)** Boxplots showing the different methylation levels between normal and tumor tissues in pan-cancer. * means p<0.05, *** means p<0.001, and **** means p<0.0001.

Investigation into the post-translational modifications (PTMs) identified the significant fold change of KLF5 methylation values across pan-cancer (**Figure 5A**). Bubble plot illustrating the correlation between KLF5 mRNA expression and DNA methylation levels in various cancers, particularly in PAAD, UVM, THYM, KIRP, BLCA (**Figure 5B**). Validation with the UALCAN database further confirmed a significantly lower level of KLF 5 methylation in multiple tumor tissues including BLCA, HNSC, KIRC, KIRP, LUAD, LUSC, PAAD, THCA, UCEC (**Figure 5C**), implicating KLF5 PTMs in tumor-related regulatory mechanisms.

### 3.3 Correlation analysis of the tumor microenvironment and immune cell infiltration

**Figure 6:**
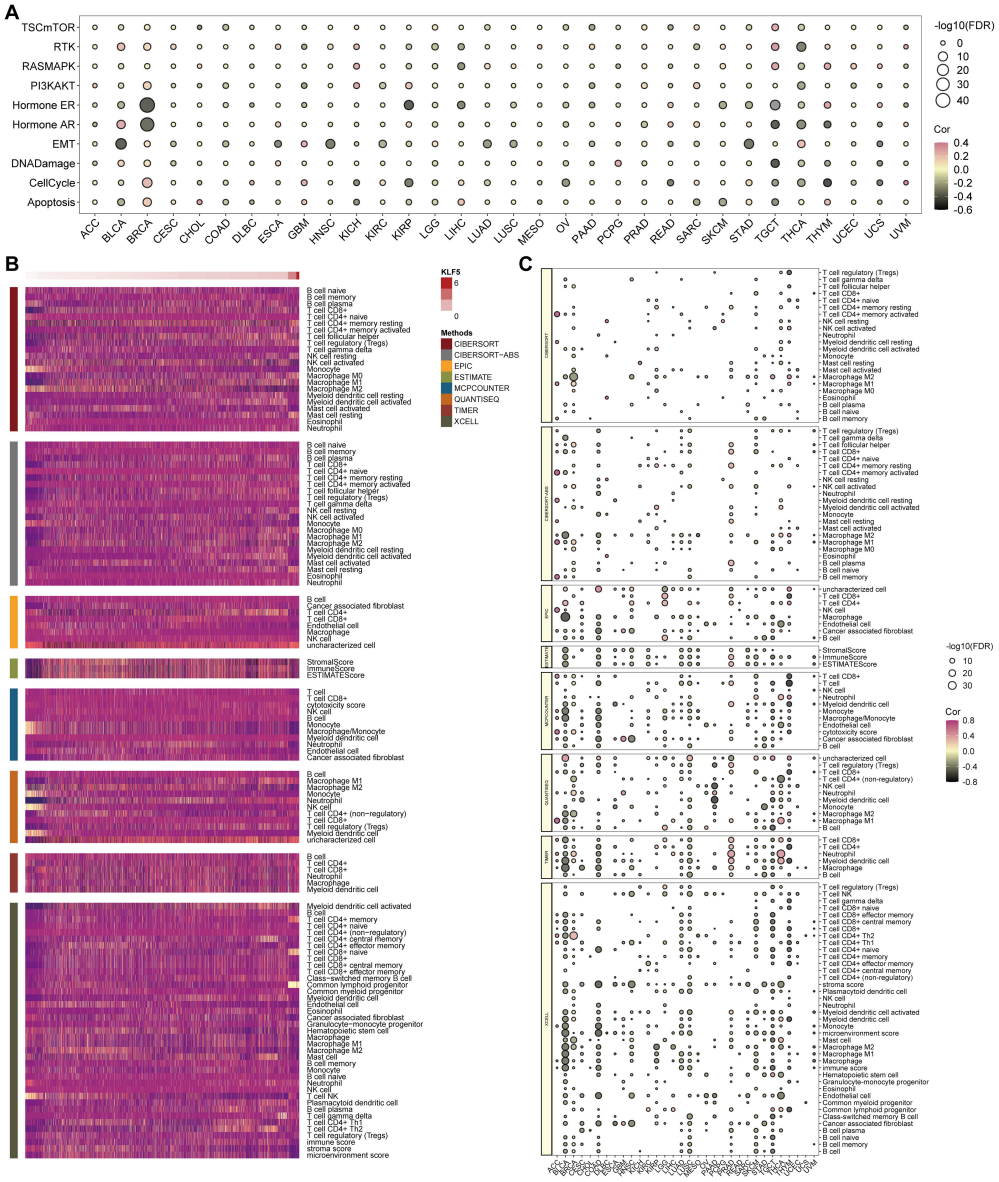
Association between KLF5 expression and cancer-related pathways and immune activity. **(A)** Bubble plots showing the correlation between KLF5 mRNA expression and classic cancer-related gene set activity in TCGA pan-cancer analysis. **(B)** Heatmap of the tumor microenvironment scores calculated by different algorithms. **(C)** Heatmap of the correlations between KLF5 and immune checkpoints in each tumor type (FDR, false discovery rate.

The relationship between KLF5 expression and the tumor immune landscape was assessed across diverse malignancies. Results from the analysis indicated that higher KLF5 levels correlated with greater infiltration of most immune cell types, as confirmed by several analytical approaches (**Figure 6A**). This correlation was universally detected across all specific cancer types investigated (**Figures 6B, C**).

### 3.4 Correlation analysis of tumor stemness and immunogenicity

**Figure 7:**
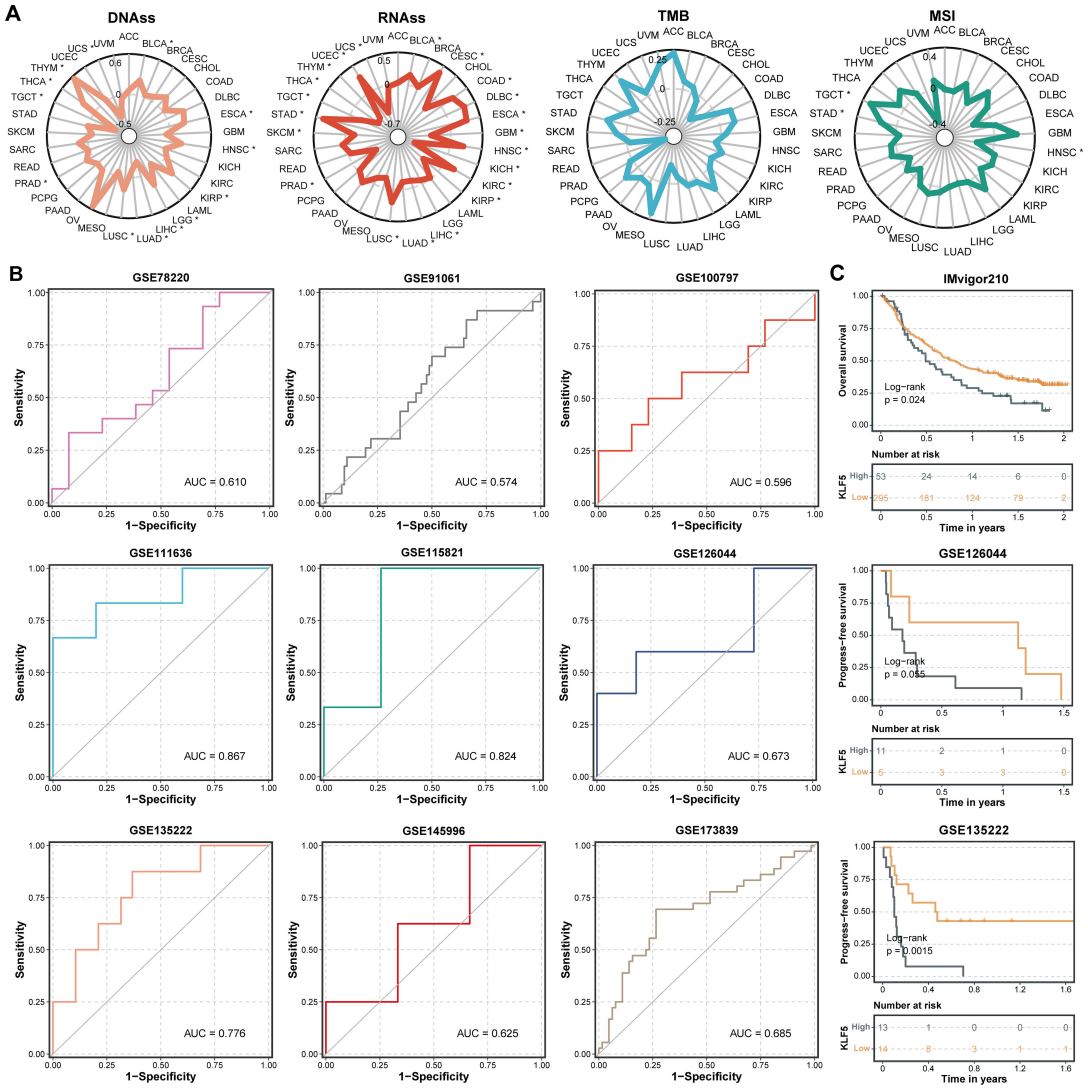
The correlations between KLF5 expression and stemness and immunogenicity, as well as the predictive efficacy of KLF5 in immunotherapy cohorts. **(A)** The correlations between KLF5 expression and RNAss, DNAss, TMB, and MSI (* p < 0.05). * means p<0.05. **(B)** The ROC curve analyses of predicting immunotherapy efficacy based on KLF5 expression in pan-cancer immunotherapy cohorts. **(C)** Kaplan–Meier survival analysis showing the prognostic role of KLF5 in pan-cancer immunotherapy cohorts.

The relationship between tumor stemness, immunogenicity, and cancer progression was evaluated across cancer types. KLF5 expression demonstrated distinct correlations with these parameters. A pronounced positive association was observed between KLF5 expression and RNA-based stemness scores (RNAss) and DNA-based stemness scores (DNAss) in most malignancies. In contrast, only a few of cancer types demonstrated correlations on microsatellite instability (MSI), and none exhibited statistically significant links between KLF5 expression and tumor mutational burden (TMB). (**Figure 7A**).

To validate the predictive capacity of KLF5 in tumor immunotherapy, we performed ROC analysis of pan-cancer immunotherapy cohorts from the GEO database, demonstrating its modest predictive efficacy for immunotherapeutic response (**Figure 7B**). Furthermore, K–M survival analysis in three independent immunotherapy cohorts, including IMvigor210 (urothelial carcinoma), GSE126044 (non-small cell lung cancer), and GSE135222 (melanoma and lung cancer), consistently revealed that patients with low KLF5 expression exhibited improved survival outcomes following immunotherapy (**Figure 7C**).

### 3.5 The validation of KLF5 as a promising biomarker

**Figure 8:**
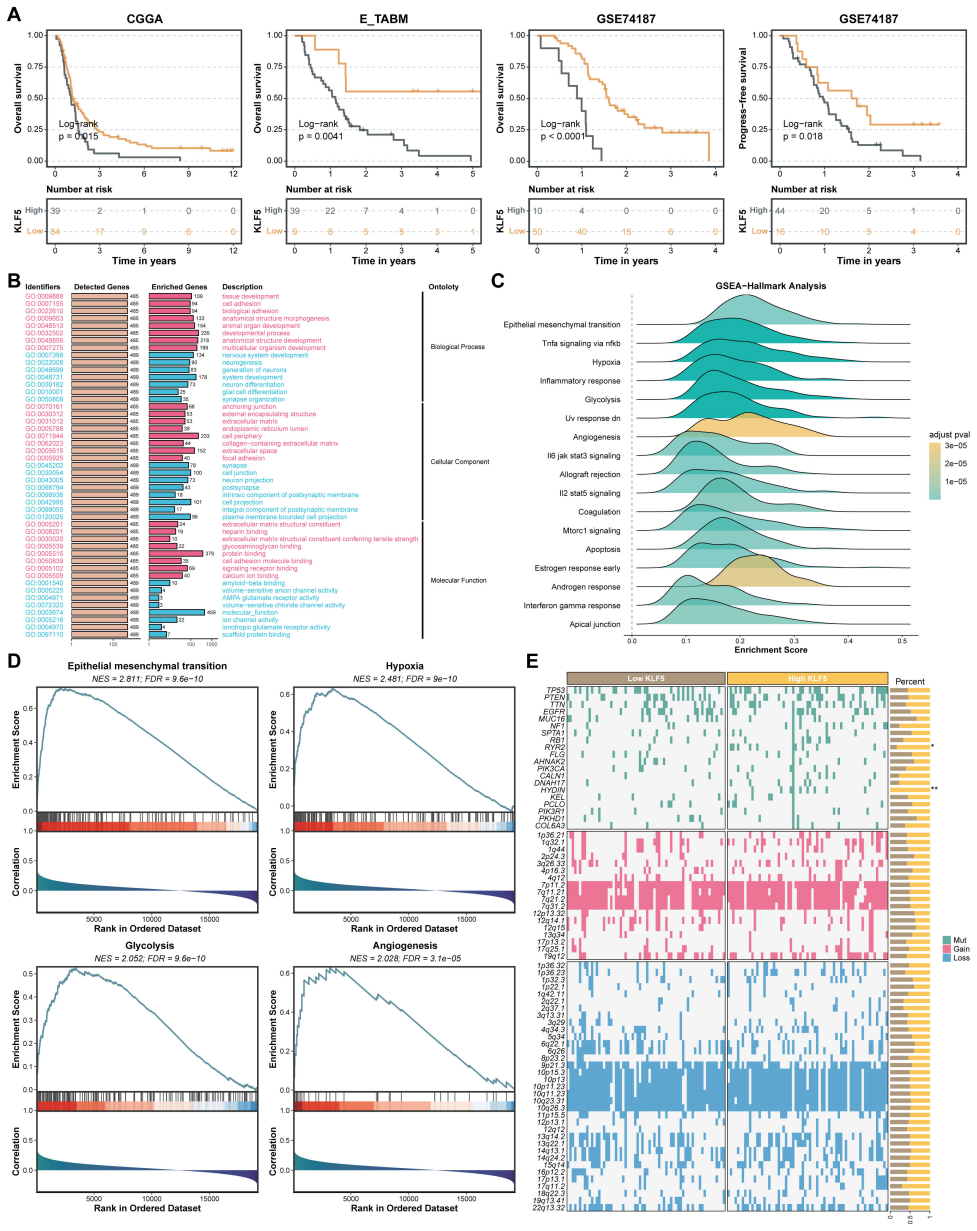
The clinical relevance of KLF5 in GBM. **(A)** Kaplan–Meier survival analysis of GBM patients stratified by KLF5 expression levels. **(B)** Enrichment analyses showing distinct biological functions and signaling pathway activity based on KLF5 expression. **(C, D)** Hallmark pathway enrichment analyses showing distinct biological functions and signaling pathway activity between KLF5 high- and low-expression. **(E)** The mutation differences based on KLF5 high- and low-expression in TCGA-GBM cohort. * means p<0.05, and ** means p<0.01.

Based on the preceding pan-cancer analysis, we investigated the potential of KLF5 as a diagnostic or prognostic biomarker in specific tumors. Evaluation of three independent glioblastoma (GBM) cohorts (CGGA, E_TABM, GSE74187) demonstrated that elevated KLF5 expression was significantly linked to poorer OS and PFS in patients (**Figure 8A**). Gene Ontology (GO) enrichment analysis further indicated that KLF5 was significantly involved in biological processes related to tissue development, cell junction organization, and nervous system development (**Figure 8B**). The result of enrichment analysis revealed distinct activation of biological functions and signaling pathways between KLF5 high- and low-expression groups (**Figures 8C, D**). Subsequent comparative analysis of differentially expressed genes in the TCGA-GBM cohort identified significant enrichment of mutations in RYR2 and HYDIN among tumors with high KLF5 expression (**Figure 8E**).

### 3.6 KLF5 knockdown could inhibit tumor progression *in vitro*

**Figure 9:**
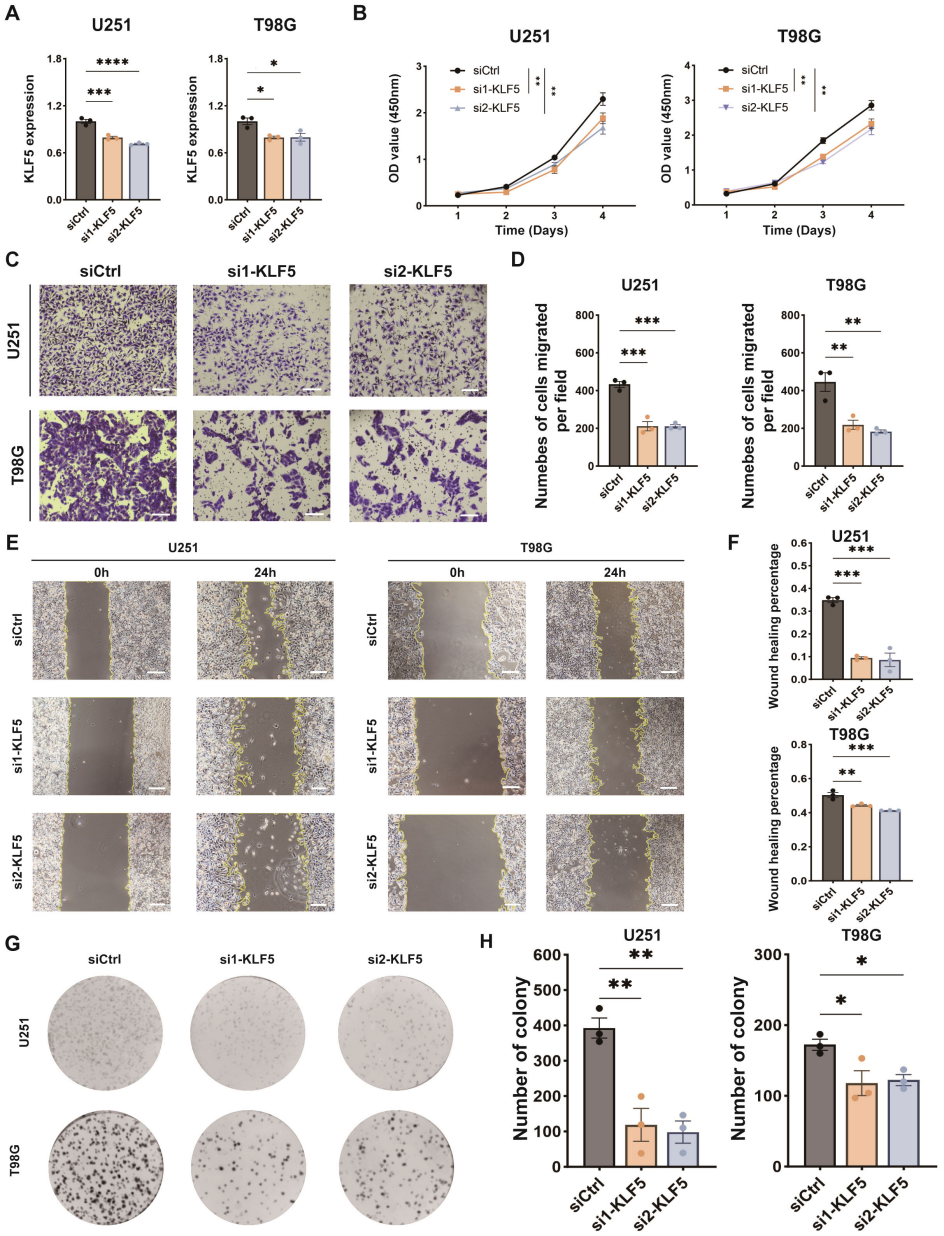
Experimental validations of KLF5 in GBM. **(A)** RT-qPCR verify the knockdown efficacy of KLF5 in GBM cell lines. **(B)** CCK-8 assays confirm KLF5 knockdown inhibits the cell viability in GBM cell lines. **(C)** Transwell assays confirm KLF5 knockdown inhibits the cell migration in GBM cell lines. **(D)** Bar plots showing the number of cells migrated per field in each group. **(E)** Wound healing assays confirm KLF5 knockdown inhibits the cell migration in GBM cell lines. **(F)** Bar plots showing the wound healing percentage in each group. **(G)** Colony formation assays confirm KLF5 knockdown inhibits the cell viability in GBM cell lines. **(H)** Bar plots showing the number of colonies in each group. * means p<0.05, ** means p<0.01, *** means p<0.001, and **** means p<0.0001.

To investigate the functional impact on GBM tumor cells of KLF5, we conduct *in vitro* validation by using GBM cell lines. We performed KLF5 knockdown in two GBM cell lines, U251 and T98G. siRNAs targeting KLF5 mRNA were transfected into cells and their knockdown efficiency was validated (**Figure 9A**). CCK-8 assays demonstrated that KLF5 silencing significantly suppressed the proliferative capacity of both U251 and T98G cells (**Figure 9B**). Furthermore, transwell invasion assays and wound healing assays indicated that KLF5 knockdown markedly reduced the migratory potential of these cells (**Figures 9C-F**). A colony formation assay further revealed a notable impairment of cell viability and clonogenic ability upon KLF5 knockdown (**Figures 9G, H**).

**Figure 10:**
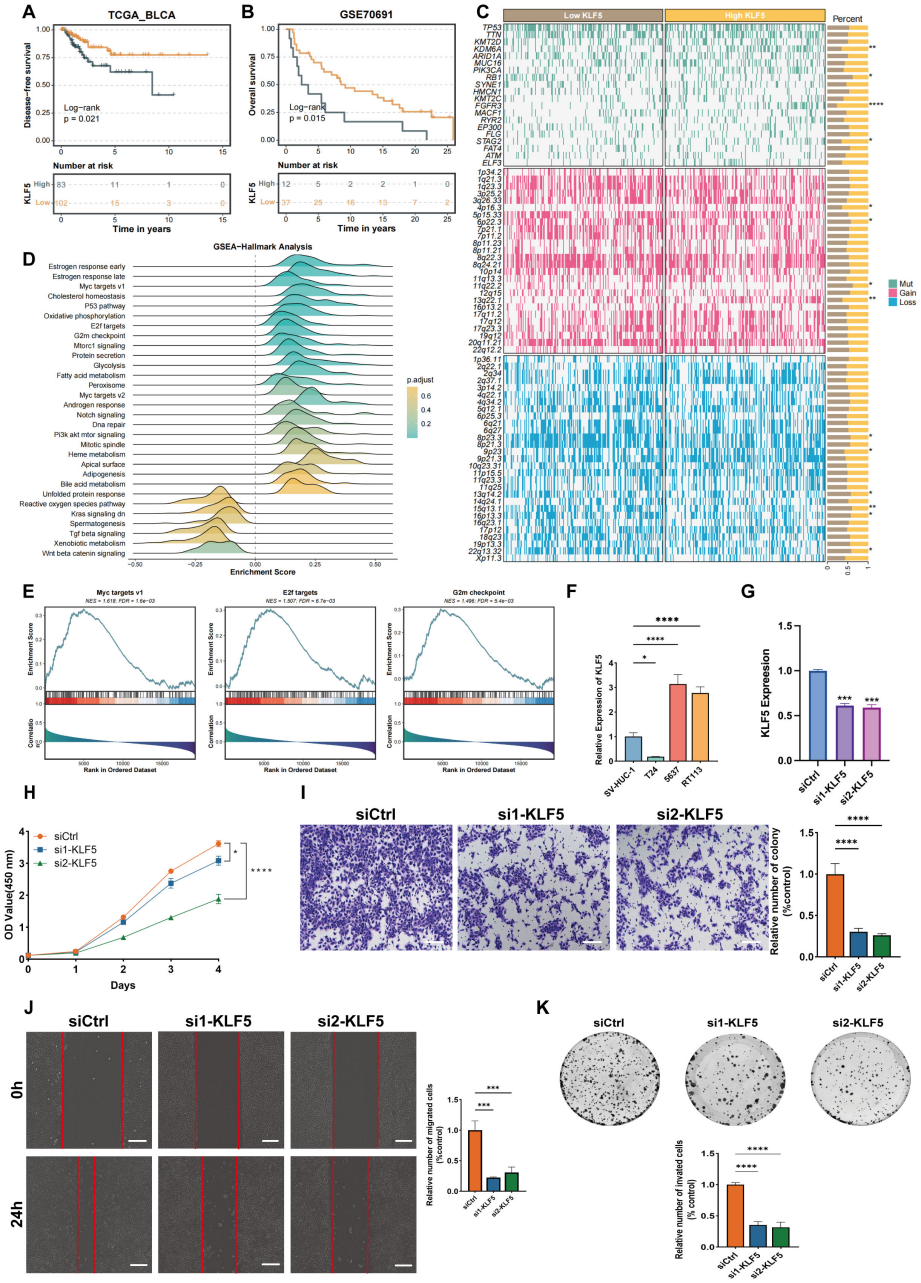
The clinical relevance and experimental validations of KLF5 in bladder cancer. **(A, B)** Kaplan–Meier survival analysis of bladder cancer patients stratified by KLF5 expression levels. **(C)** The mutation differences based on KLF5 high- and low-expression in TCGA-BLCA cohort. **(D, E)** Hallmark pathway enrichment analyses showing distinct biological functions and signaling pathway activity between KLF5 high- and low-expression. **(F)** RT-qPCR showing the mRNA expression level of KLF5 in bladder cell lines. **(G)** RT-qPCR verify the knockdown efficacy of KLF5 in bladder cancer cell line. **(H)** CCK-8 assays confirm KLF5 knockdown inhibits the cell viability in bladder cancer cell line. **(I)** Transwell assays confirm KLF5 knockdown inhibits the cell migration in bladder cancer cell line. **(J)** Wound healing assays confirm KLF5 knockdown inhibits the cell migration in bladder cancer cell line. **(K)** Colony formation assays confirm KLF5 knockdown inhibits the cell viability in bladder cancer cell line. * means p<0.05, ** means p<0.01, *** means p<0.001, and **** means p<0.0001.

To further validate its role in pan-cancer, we then focused on the role of KLF5 in bladder cancer. We performed K–M survival analysis and we found that higher KLF5 expression was correlated with unfavorable prognosis in bladder cancer patients (**Figures 10A, B**). Besides, we found that elevated KLF5 expression level was related to higher KDM6A, FGFR3, and STAG2 mutations, as well as higher 4p16.3 and 13q22.1 gain ratios (**Figure 10C**). Additionally, enrichment analysis indicated that KLF5 participated in several oncogenic pathways such as Myc targets v1, E2f targets, and G2m checkpoint (**Figures 10D, E**). We further conducted RT-qPCR to assess the mRNA expression of KLF5, and the result showed that KLF5 was upregulated in bladder cancer cell lines like 5637 and RT113 (**Figure 10F**). Since 5637 possessed the highest KLF5 expression level, we conducted KLF5 knockdown using siRNA in this bladder cancer cell line (**Figure 10G**). Consistent with the verification results obtained in GBM cell lines, we found that KLF5 knockdown significantly inhibited the ability of bladder cancer cell proliferation and migration (**Figures 10H-K**). These results indicated that KLF5 knockdown could inhibit tumor progression *in vitro*.

## 4 Discussion

Cancer poses a major global public health challenge (1). While existing research has extensively explored molecular mechanisms and pathways in individual cancer types, the emergence of multi-omics databases now enables systematic pan-cancer analyses (38–41). This approach provides a unified framework to investigate specific molecules across malignancies, allowing comprehensive characterization of their transcriptional profiles, expression patterns, post-translational modifications, and pathway activities (42, 43). The fundamental value of pan-cancer analysis lies in its capacity to distinguish universally conserved oncogenic drivers from context-dependent mechanisms, simultaneously revealing both shared therapeutic vulnerabilities and the origins of tissue-specific oncogenic heterogeneity (44).

Within this framework, a focused pan-cancer analysis of KLF5 is warranted. While previous studies have established KLF5’s context-dependent roles—functioning as a potent oncogene in carcinomas such as colorectal (19), yet exhibiting tumor-suppressive properties in specific contexts like breast cancer and prostate cancer (22, 24, 25, 45)—these fragmented findings necessitate a holistic reinterpretation. A pan-cancer analysis is critical to resolve this apparent paradox. It allows for the systematic determination of whether KLF5’s dual nature is dictated by tissue-of-origin, specific mutational backgrounds. By mapping its genomic alteration spectra, expression profiles, and associated clinical outcomes across the cancer spectrum, we can delineate a definitive functionality map for KLF5.

We observed notably high expression of KLF5 in normal tissues such as the gastrointestinal epithelium, urothelium, lung and skin. Similarly, studies of cell line databases consistently showed increased KLF5 expression in gastrointestinal, bladder, lung, and head and neck cancers. However, analysis of tumor and matched normal tissues from TCGA database demonstrated that while elevated KLF5 expression was observed in gastrointestinal tumors, its expression was conversely lower in several other cancers, including breast cancer, prostate cancer, and kidney cancer. This pattern may suggest a context-dependent role for KLF5 expression in tumorigenesis. A comprehensive cancer survival analysis demonstrated a strong correlation between KLF5 expression and patient prognosis, underscoring its role as a prognostic biomarker. Analysis of KLF5 copy number variation revealed two major patterns: heterozygous amplification, primarily in gastrointestinal, lung adenocarcinoma, and bladder cancers, and heterozygous deletion with a broader distribution across multiple cancer types. It indicates that determining whether KLF5 acts as an oncogene requiring amplification or a context-dependent tumor suppressor susceptible to loss is essential for understanding its pathobiology in a given tumor (46). In addition, we analyzed the pan-cancer profile of PTMs of KLF5 and identified significant DNA hypomethylation across multiple cancer types. However, previous studies indicates the hypermethylation of KLF5 may promote caner (47, 48). Further characterization of specific methylation sites will help elucidate the regulatory role and functional impact of KLF5 methylation in pan-cancer contexts (49, 50).

GBM, remains the most aggressive primary brain tumor with a dismal prognosis. a major bottleneck in glioma treatment lies in the lack of specific targeted therapeutic targets: existing targeted drugs rarely achieve durable efficacy due to the blood-brain barrier, tumor heterogeneity, and rapid development of drug resistance (51). Similarly, bladder cancer, particularly muscle-invasive bladder cancer, faces significant clinical challenges. Although immune checkpoint inhibitors have improved outcomes for some patients, most cases still require radical cystectomy, which severely impairs quality of life (52). Against this backdrop, identifying novel molecular targets and prognostic biomarkers for glioma and bladder cancer is critical to breaking through current treatment bottlenecks. We further selected various cell lines of GBM and bladder cancer for functional cellular assays, and the results indicated that KLF5 could serve as a potential biomarker for glioma and bladder cancer.

Currently, the research and development of KLF5 inhibitors presents diversified characteristics in terms of structural types and mechanisms of action. Among small-molecule inhibitors that directly target KLF5, ML264 and its optimized derivative SR18662 are the representative compounds that first came into research view (53, 54). In addition, Pterosin B derived from natural products can not only inhibit KLF5 expression, but also improve cognitive impairment and alleviate myocardial hypertrophy simultaneously, demonstrating multi-target therapeutic potential (55, 56). At the preclinical research and translational application level, KLF5 inhibitors have shown broad tumor type adaptability and potential for combination therapy. Despite the phased breakthroughs achieved in the research and development of KLF5 inhibitors, many challenges remain: some compounds have problems such as insufficient *in vivo* activity and poor targeting; the physiological functions of KLF5 in normal tissues may also lead to off-target toxicity of inhibitors; and the lineage plasticity of tumor cells may trigger acquired resistance to KLF5 inhibitors. Therefore, in-depth analysis of the tissue-specific regulatory mechanism of KLF5, development of highly selective inhibitors, and exploration of multi-target combination strategies will become the core directions to promote the transition of KLF5-targeted therapy from basic research to clinical application.

The study has particular limitations that should be noted. First, most of the enrolled datasets were derived publicly, additional in-house samples and cohorts are necessary to be replenished to validate our findings. Second, although we assessed that KLF5 was vital in GBM and bladder cancer progression, the lack of *in vivo* validation using animal models limits the translation of our *in vitro* findings to clinical settings, and in-depth mechanisms based on this phenomenon should be conducted and the interaction between KLF5 and tumor microenvironment are crucial to be explored. Third, the potential application of KLF5 inhibitors into clinical practice need to be verified, either alone or in combination with existing therapies. These efforts will further consolidate the role of KLF5 as a prognostic biomarker and therapeutic target, facilitating its translation into clinical practice.

## 5 Conclusion

Collectively, we provide thorough exploration of KLF5 across various cancers. systematically characterizing its expression patterns, genomic alterations, and clinical relevance across diverse human cancers. We experimentally validated its potent oncogenic functions in GBM and bladder cancer cell lines. Our work confirms that KLF5 plays distinct, context-dependent roles in different tumor types, yet its targeted inhibition emerges as a consistently viable therapeutic strategy. Importantly, these findings position KLF5 as a promising molecular biomarker with the potential to guide more precise and effective treatment decisions in clinical oncology.

## Data Availability

The original contributions presented in the study are included in the article/**Supplementary Material**. Further inquiries can be directed to the corresponding authors.
