# Supplementary material for: Dissecting the role of KLF5: from tumor progression to immune interactions with emphasis on glioma and bladder cancer
Source: Front Immunol. 2026 Jan 8;16:1730356. doi: 10.3389/fimmu.2025.1730356 (PMC12823895; doi:10.3389/fimmu.2025.1730356)
Supplement: Supplementary file 1 [file DataSheet1.pdf]

Supplementary Material

1 Supplementary Tables

Supplementary Table S1. Summary of TCGA and GTEx sample sizes of different tumor types in this study.

Abbreviations: ACC, Adrenocortical Cancer; BLCA, Bladder Cancer; BRCA, Breast Cancer; CESC, Cervical Cancer; CHOL, Bile Duct Cancer; COAD, Colon Adenocarcinoma; DLBC, Large B-cell Lymphoma; ESCA, Esophageal Cancer; GBM, Glioblastoma; HNSC, Head and Neck Squamous Cell Carcinoma; KICH, Kidney Chromophobe; KIRC, Kidney Renal Clear Cell Carcinoma; KIRP, Kidney Renal Papillary Cell Carcinoma; LAML, Acute Myeloid Leukemia; LGG, Lower Grade Glioma; LIHC, Liver Hepatocellular Carcinoma; LUAD, Lung Adenocarcinoma; LUSC, Lung Squamous Cell Carcinoma; MESO, Mesothelioma; OV, Ovarian Cancer; PAAD, Pancreatic Cancer; PCPG, Pheochromocytoma & Paraganglioma; PRAD, Prostate Adenocarcinoma; READ, Rectum Adenocarcinoma; SARC, Sarcoma; SKCM, Skin Cutaneous Melanoma; STAD, Stomach Adenocarcinoma; TGCT, Testicular Cancer; THCA, Thyroid Cancer; THYM, Thymoma; UCEC, Uterine Corpus Endometrial Carcinoma; UCS, Uterine Carcinosarcoma; UVM, Ocular melanomas.

Tumor type	TCGA (Tumor)	TCGA (Normal)	GTEx (Normal)
ACC	79	0	258
BLCA	406	19	21
BRCA	1101	113	459
CESC	306	3	19
CHOL	35	9	0

COAD	455	41	779
DLBC	48	0	929
ESCA	163	11	1445
GBM	153	5	2642
HNSC	504	44	0
KICH	65	25	89
KIRC	532	72	89
KIRP	290	32	89
LAML	150	0	0
LGG	513	0	2642
LIHC	371	50	226
LUAD	516	59	578
LUSC	501	49	578
MESO	87	0	0
OV	376	0	180
PAAD	179	4	328

PCPG	181	3	0
PRAD	498	52	245
READ	165	10	779
SARC	260	2	0
SKCM	471	1	1809
STAD	375	32	359
TGCT	134	0	361
THCA	512	59	653
THYM	120	2	0
UCEC	545	35	142
UCS	57	0	142
UVM	80	0	0

Supplementary Table S2. Primers for qRT-PCR detection.

KLF5	Forward	CCTGGTCCAGACAAGATGTGA
	Reverse	GAACTGGTCTACGACTGAGGC
GAPDH	Forward	CTGGGCTACACTGAGCACC
	Reverse	AAGTGGTCGTTGAGGGCAATG

Supplementary Table S3. siRNAs sequence.

Non-targeting	siCtrl	CTGTCACCACAGTAGCTTGG
KLF5	si1-KLF5	GCUCCAGAGGUGAACAAUATT
	si2-KLF5	GCAUCCACUACUGCGAUUATT
